# Supplementary material for: Facilitating two-electron oxygen reduction with pyrrolic nitrogen sites for electrochemical hydrogen peroxide production
Source: Nat Commun. 2023 Jul 22;14:4430. doi: 10.1038/s41467-023-40118-y (PMC10363113; doi:10.1038/s41467-023-40118-y)
Supplement: Supplementary file 1 — Supplementary Information [file 41467_2023_40118_MOESM1_ESM.pdf]

Supporting Information

Facilitating Two-electron Oxygen Reduction with Pyrrolic Nitrogen Sites for Electrochemical Hydrogen Peroxide Production

Wei Peng[1], *Jiaxin Liu*[1], *Xiaoqing Liu*[1], *Liqun Wang*[2]*, *Lichang Yin*[3]*, *Haotian Tan*[1], *Feng Hou*[1]*, *Ji Liang*[1]*

1 Key Laboratory of Advanced Ceramics and Machining Technology of Ministry of Education School of Materials Science and Engineering, Tianjin University, Tianjin 300072, China.

2 Applied Physics Department, College of Physics and Materials Science, Tianjin Normal University, Tianjin 300387, China.

3 Shenyang National Laboratory for Materials Science, Institute of Metal Research, Chinese Academy of Sciences, Shenyang, Liaoning 110016, China.

E-mail: wlxywlq@mail.tjnu.edu.cn (**L. Wang**), houf@tju.edu.cn (**F. Hou**), lcyin@imr.ac.cn (**L. Yin**) and liangji@tju.edu.cn (**J. Liang**)

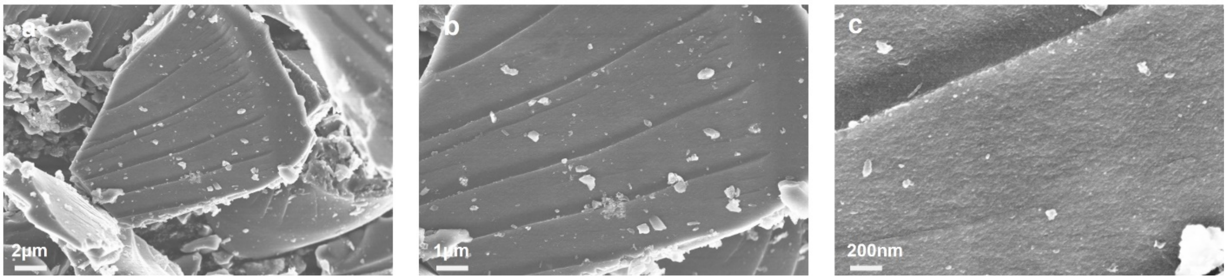

Figure S1. a-c SEM images of P-NMG-0.

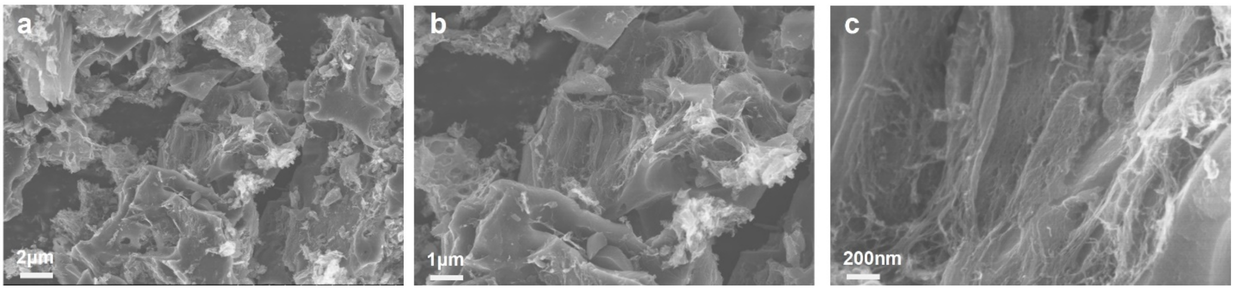

Figure S2. a-c SEM images of P-NMG-5.

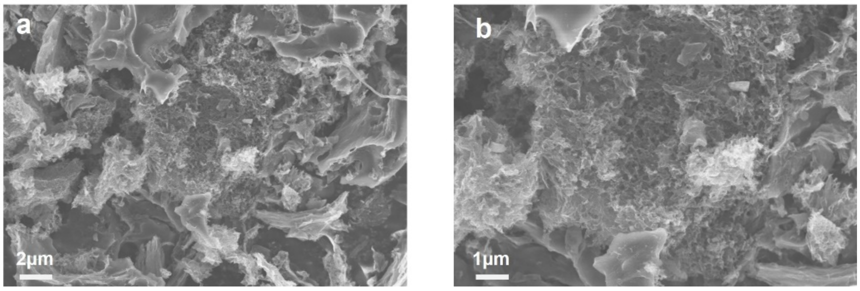

Figure S3. a-b SEM images of P-NMG-10.

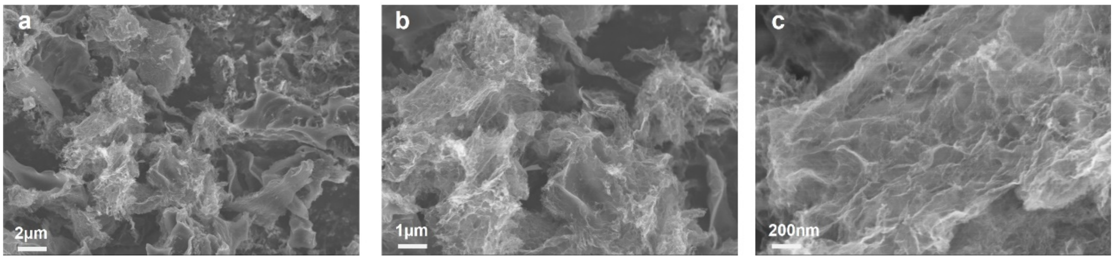

Figure S4. a-c SEM images of P-NMG-15.

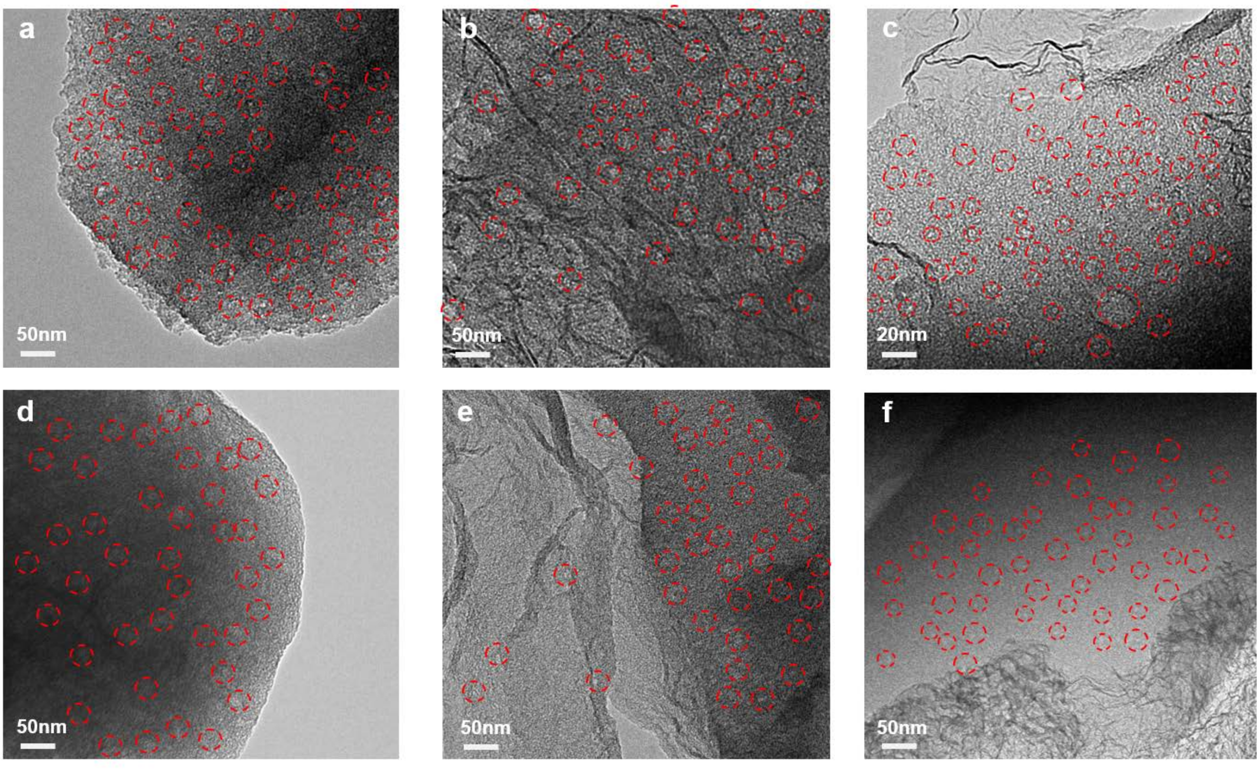

Figure S5. a-f TEM images of P-NMG-10.

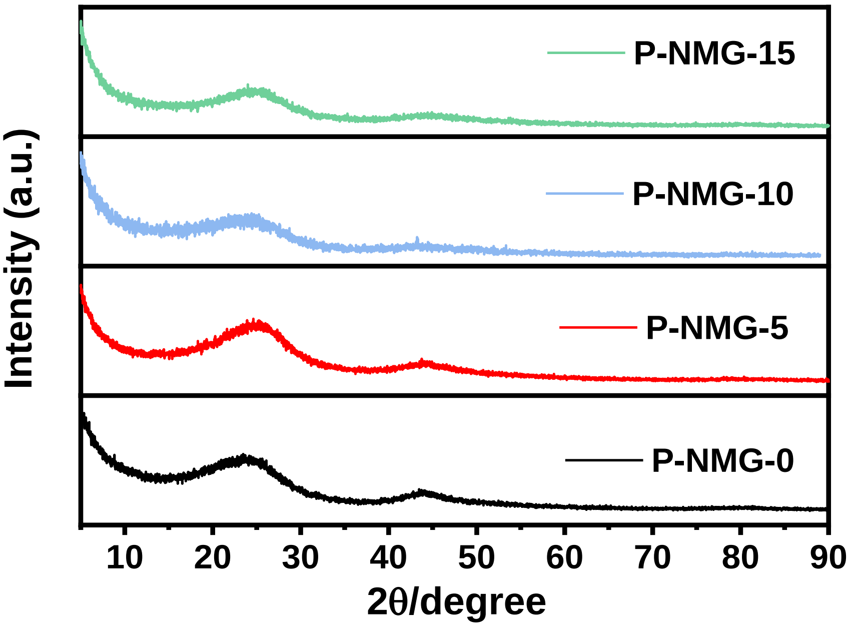

Figure S6. XRD patterns of P-NMG-0, P-NMG-5, P-NMG-10 and P-NMG-15.

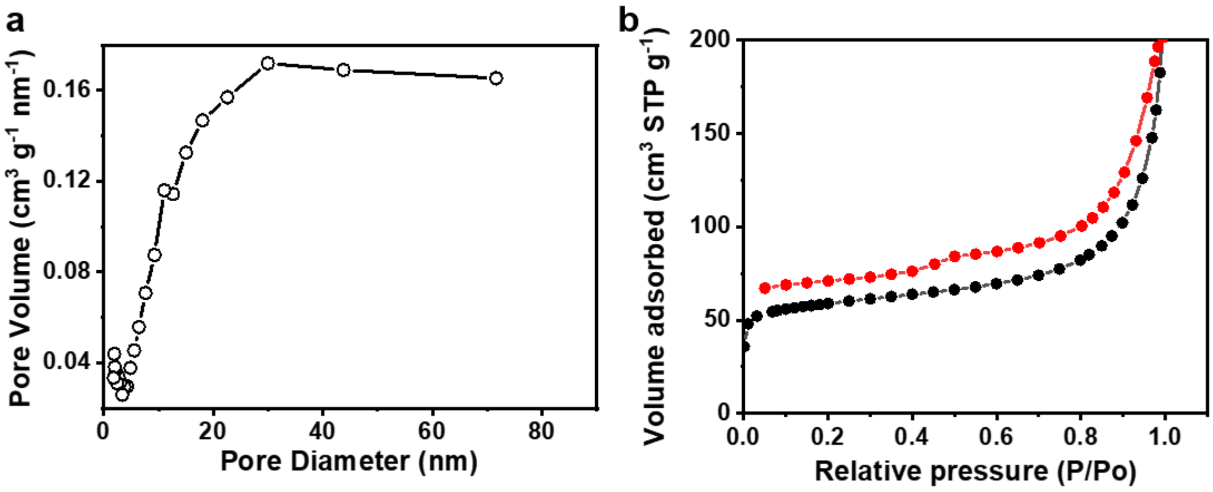

Figure S7. a Nitrogen adsorption-desorption isotherm, and **b** its corresponding pore distribution pattern of P-NMG-5.

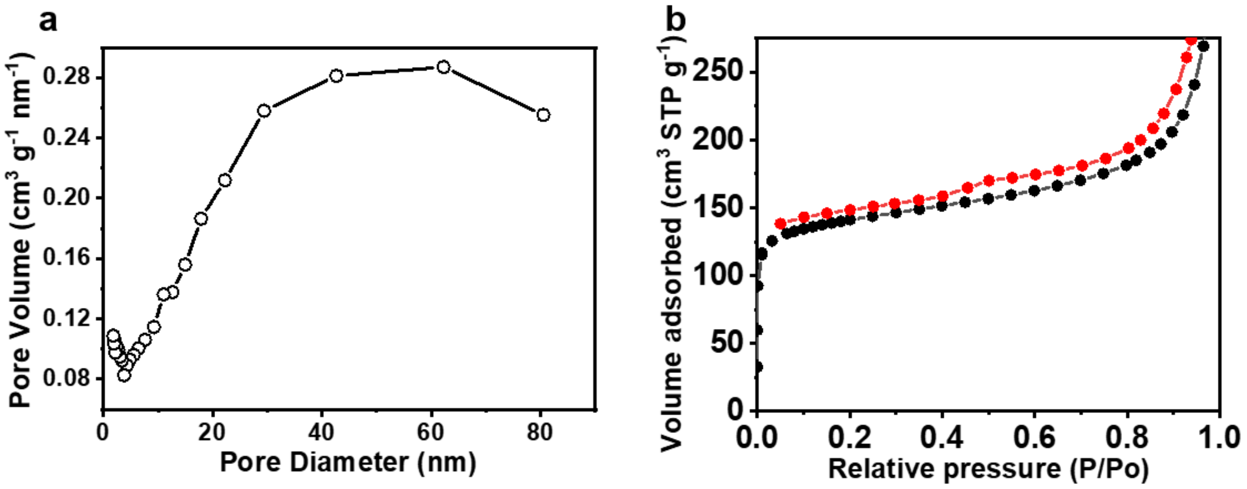

Figure S8. a Nitrogen adsorption-desorption isotherm, and **b** its corresponding pore distribution pattern of P-NMG-15.

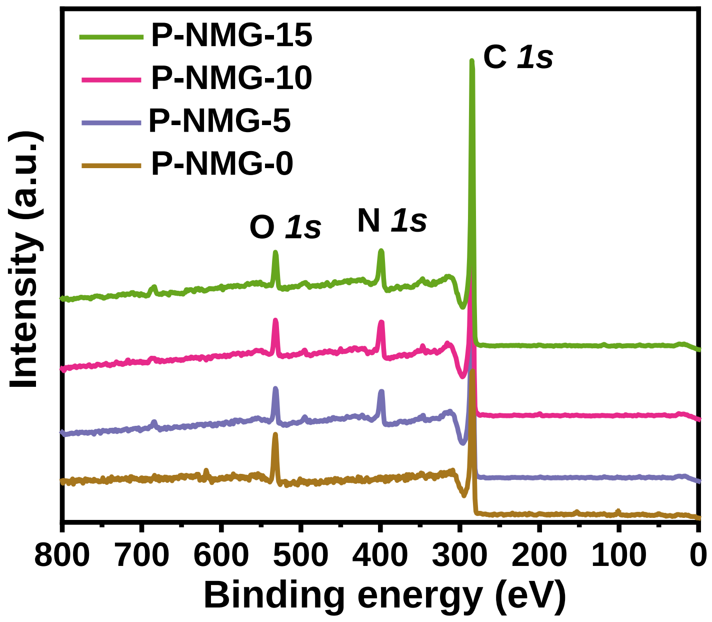

Figure S9. XPS full spectrum of P-NMG-X**.**

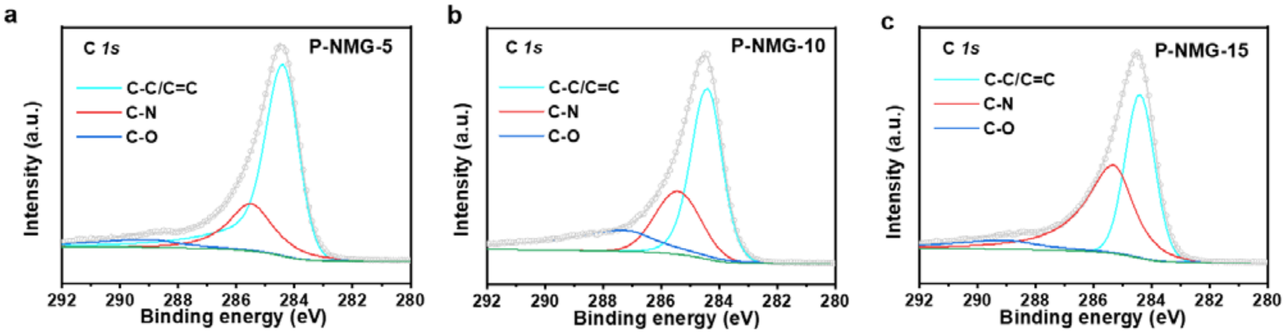

Figure S10. High-resolution C *1s* XPS spectra of **a** P-NMG-5, **b** P-NMG-10, and **c** P-NMG-15.

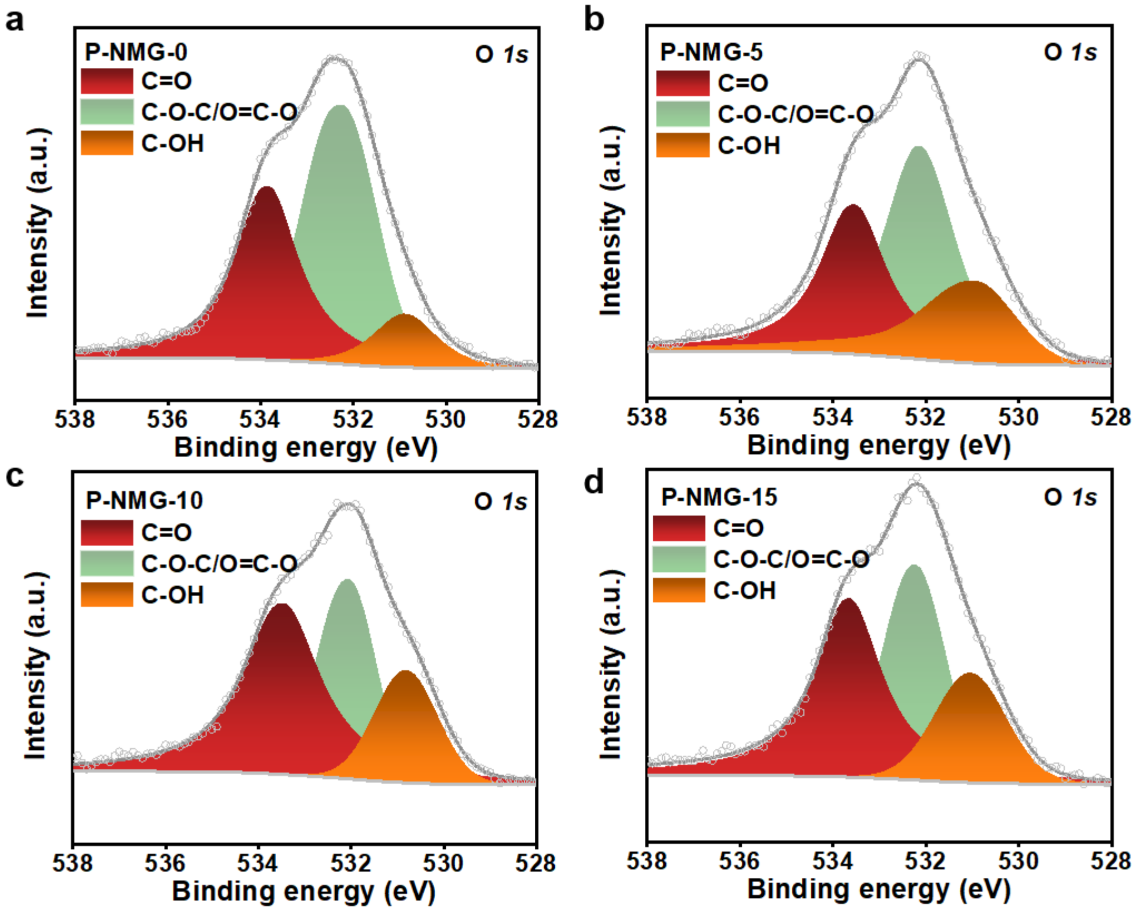

Figure S11. High-resolution O *1s* XPS spectra of **a** P-NMG-0, **b** P-NMG-5, **c** P-NMG-10, and **d** P-NMG-15.

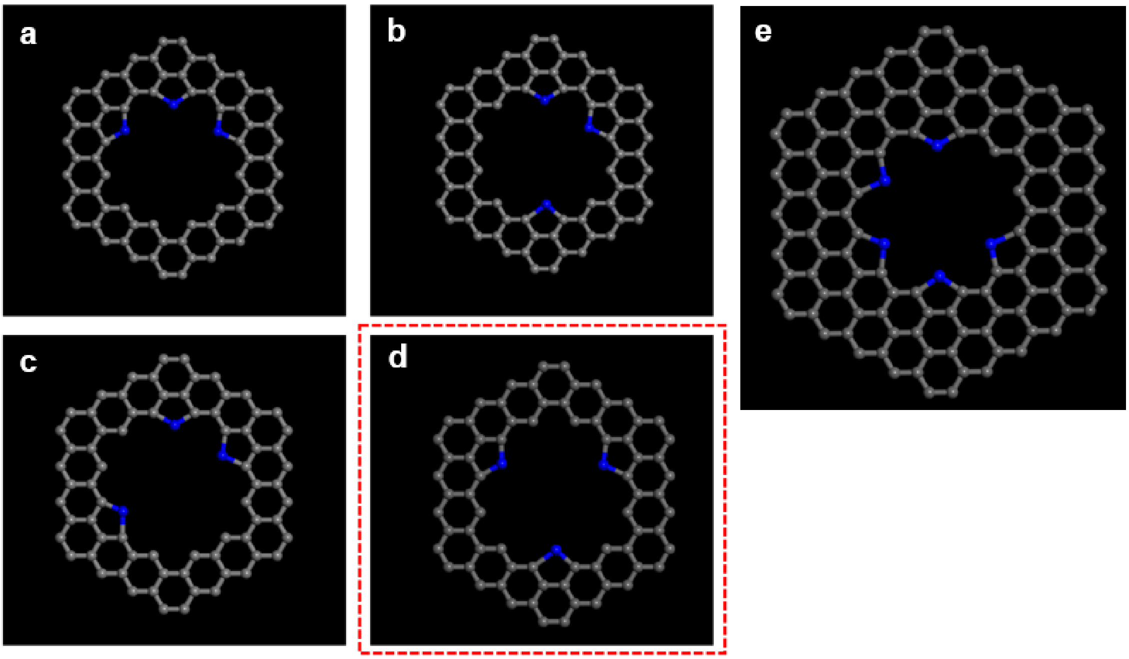

Figure S12. a-e Possible distribution of pyrrolic nitrogen atoms in different substrates.

we have constructed the material's possible structural models on the basis of the atomic proportions of C (grey balls) and N (blue balls), with the existence of such small-sized micropores. Even in an extreme scenario where each micropore (2 nm) is surrounded by only one layer of six-membered rings (**Figure S12a-d**, i.e., the most extremely porous case that may exist, with the largest possible amounts of pores on materials), three pyrrolic nitrogen atoms still exist at the edge of the pore. In this case, adjacent multiple pyrrolic nitrogen structures would form in three of the above four cases. In other cases, where the pore/carbon ratio is lower than in these extreme cases, multiple pyrrolic nitrogen configurations appear almost inevitably, as shown in **Figure S12e**. Consequently, the proposed multiple pyrrolic nitrogen configurations should almost definitely exist in our material from a statistical point of view.

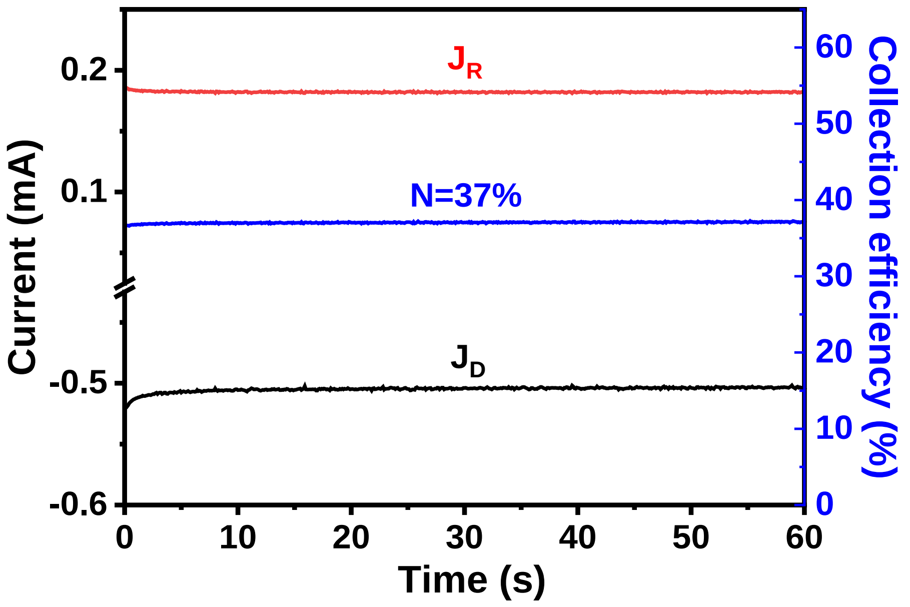

Figure S13. The collection efficiency (*N*) immersed in Ar-saturated 0.1 M PBS with 5mM of potassium ferricyanide ($K_3Fe(CN)_6$).

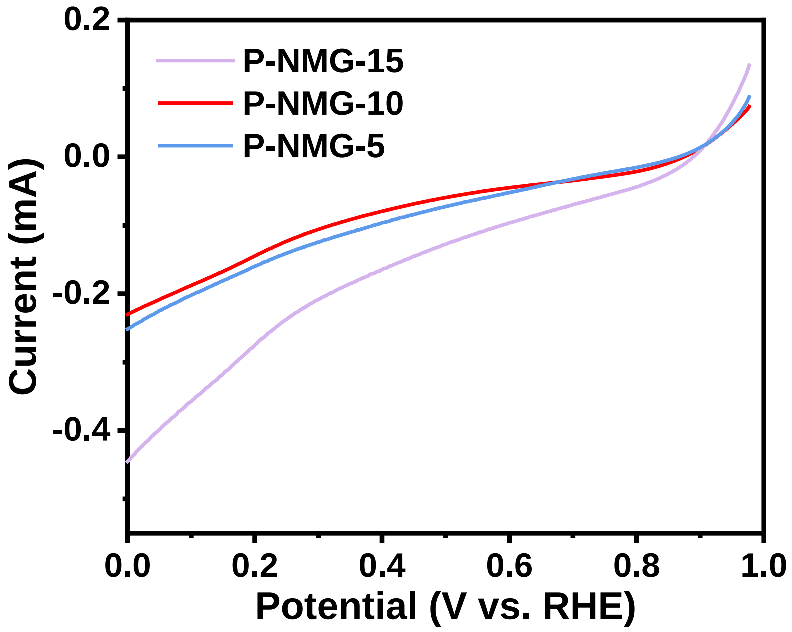

Figure S14. LSV curves of P-NMG-5, P-NMG-10 and P-NMG-15 in 0.10 M KOH containing 50 mM H_2O_2.

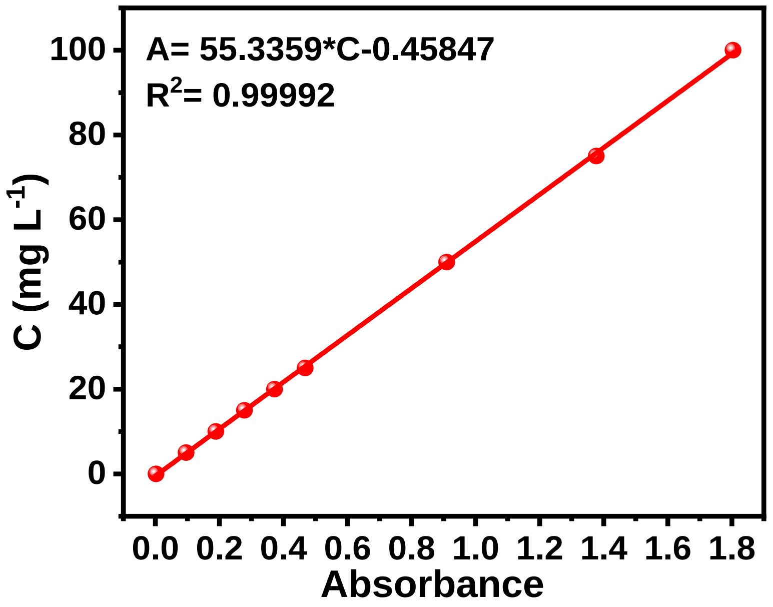

Figure S15. Corresponding standard curve.

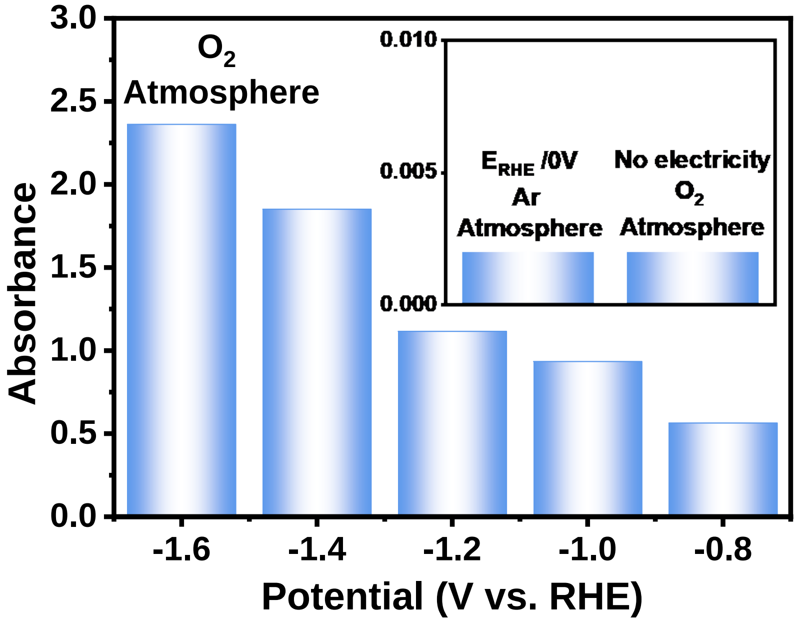

Figure S16. The blank control groups.

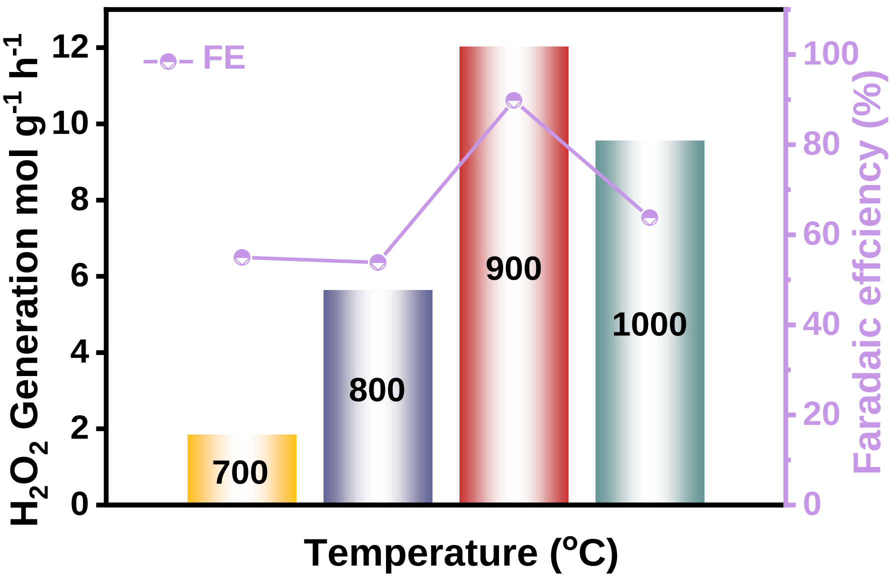

Figure S17. H_2O_2 yield of the samples obtained from different temperature treatments.

The samples obtained from different temperature treatments were tested (**Figure S14 and 15**), and it can be observed that the highest yield and FE were obtained (0 V vs. RHE) from the samples treated at 900 °C. It proved that 900 °C is the optimum annealing temperature for P-NMG-X.

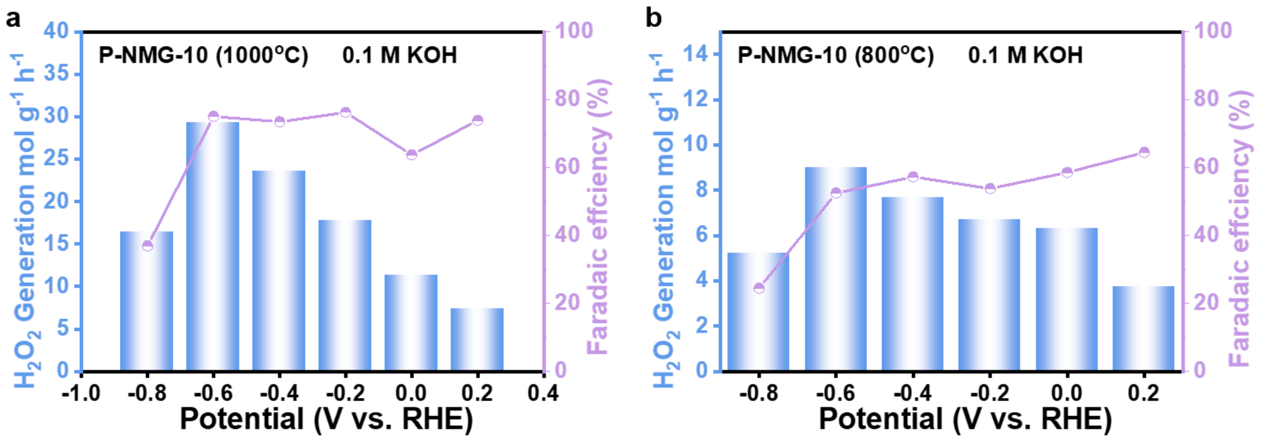

Figure S18. a The yield and FE of H_2O_2 for the samples obtained from 1000 °C. **b** The yield and FE of H_2O_2 for the samples obtained from 800 °C.

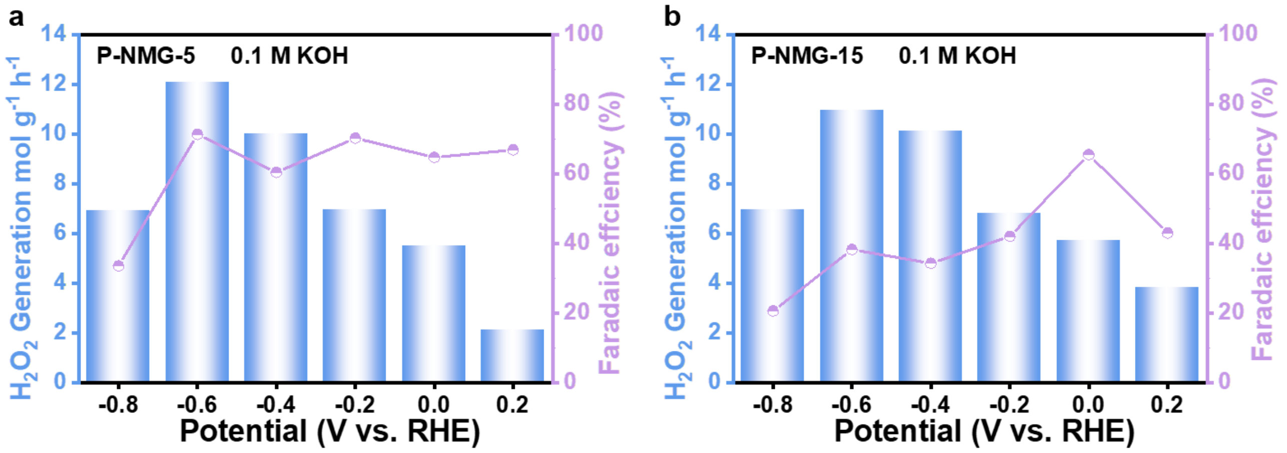

Figure S19. **a** The yield and FE of H_2O_2 for the P-NMG-5. **b** The yield and FE of H_2O_2 for the P-NMG-15.

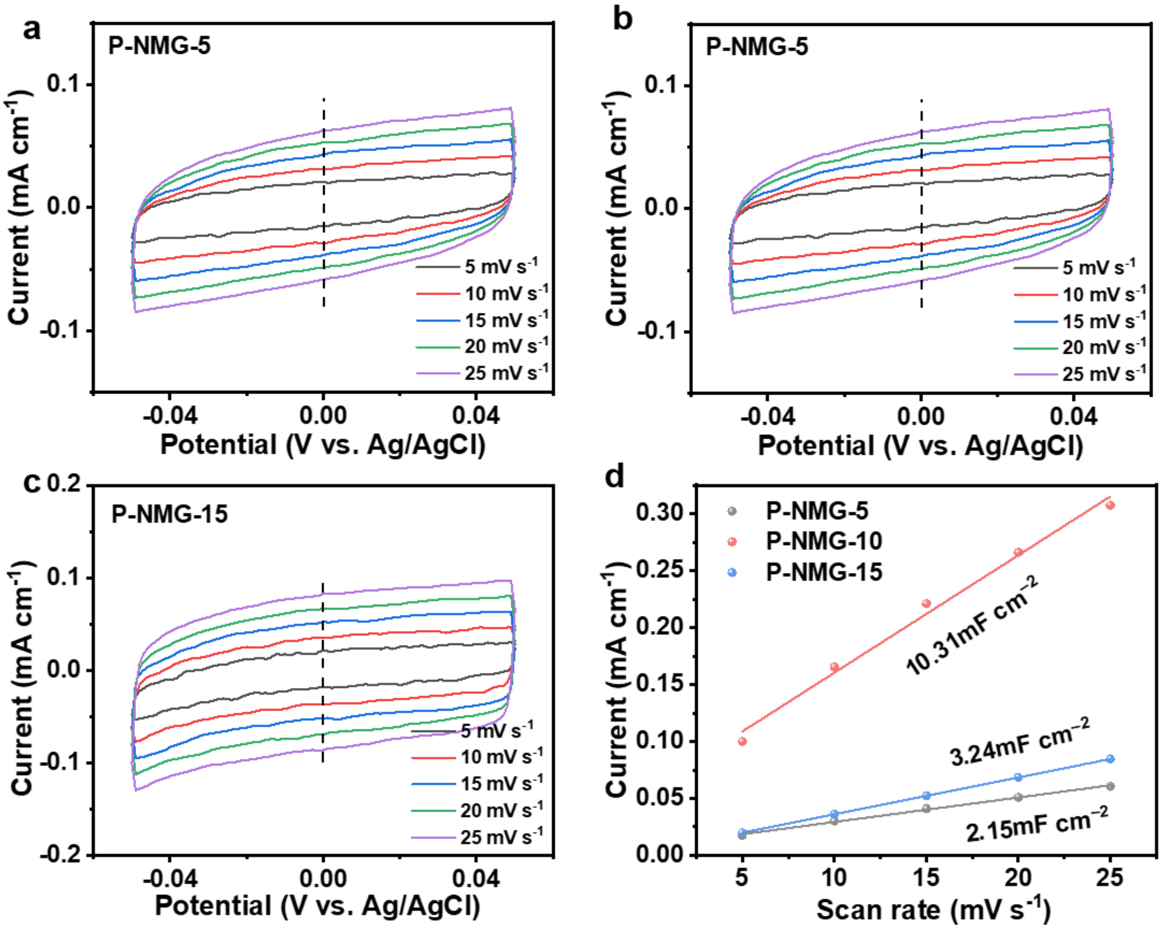

Figure S20. Determination of electrochemically active surface area. **a-c** CV curves of P-NMG-5, P-NMG-10 and P-NMG-15. **d** The C_{dl} for P-NMG-5, P-NMG-10 and P-NMG-15 are calculated to be 2.15, 3.24 and 10.31 mF cm^{-2}, respectively.

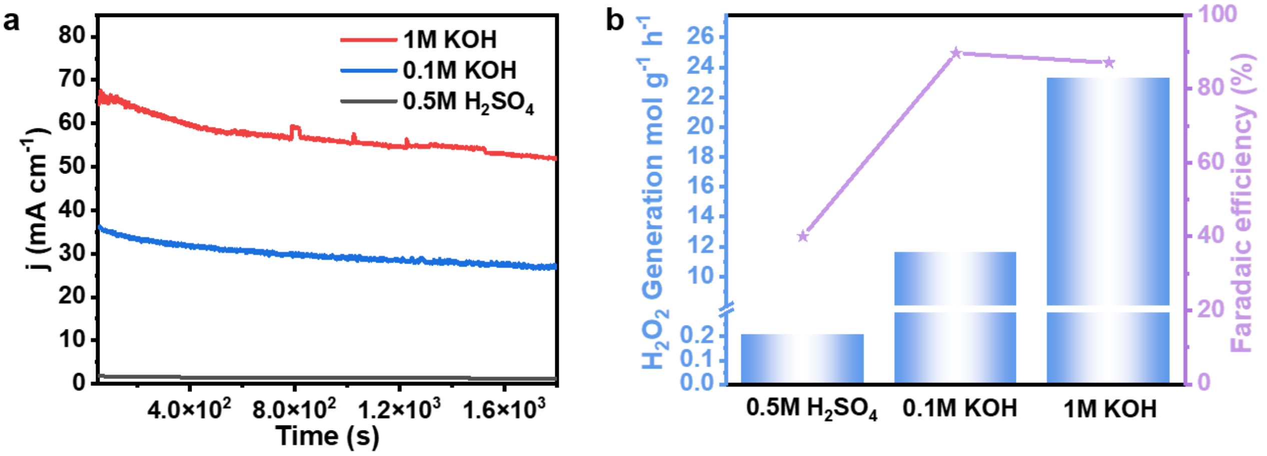

Figure S21 a Current density **b** H_2O_2 yield and FE of P-NMG-10 in 0.1 M KOH, 1 M KOH and 0.5 M H_2SO_4 electrolyte.

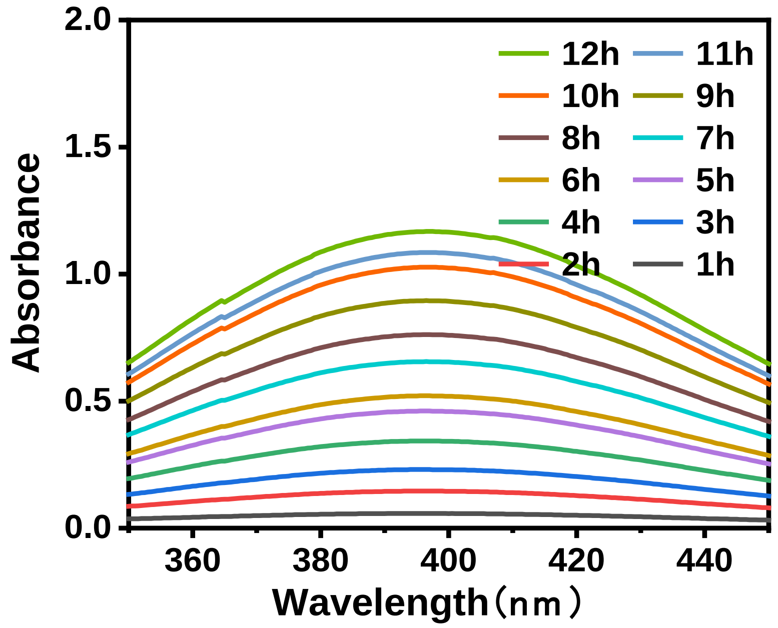

Figure S22. UV-vis spectra of electrolyte every hour.

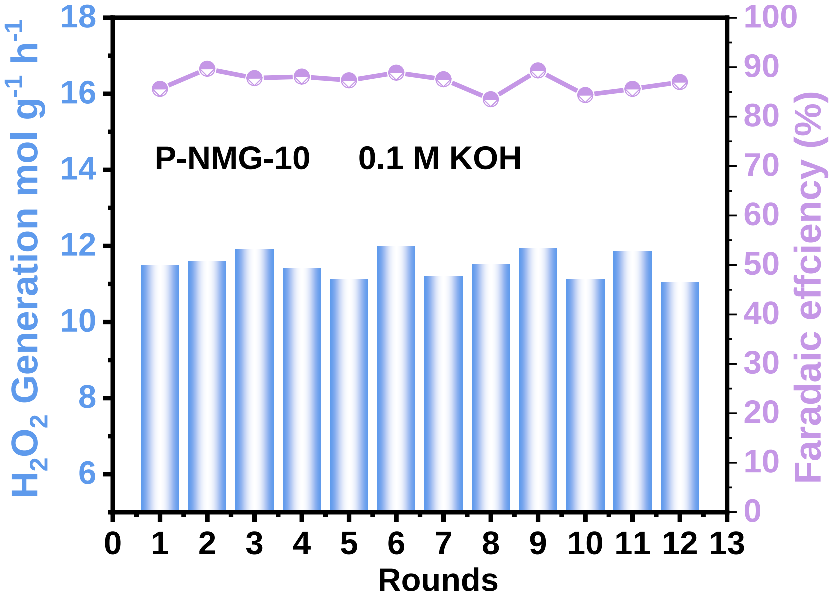

Figure S23. Yield and FE for different rounds.

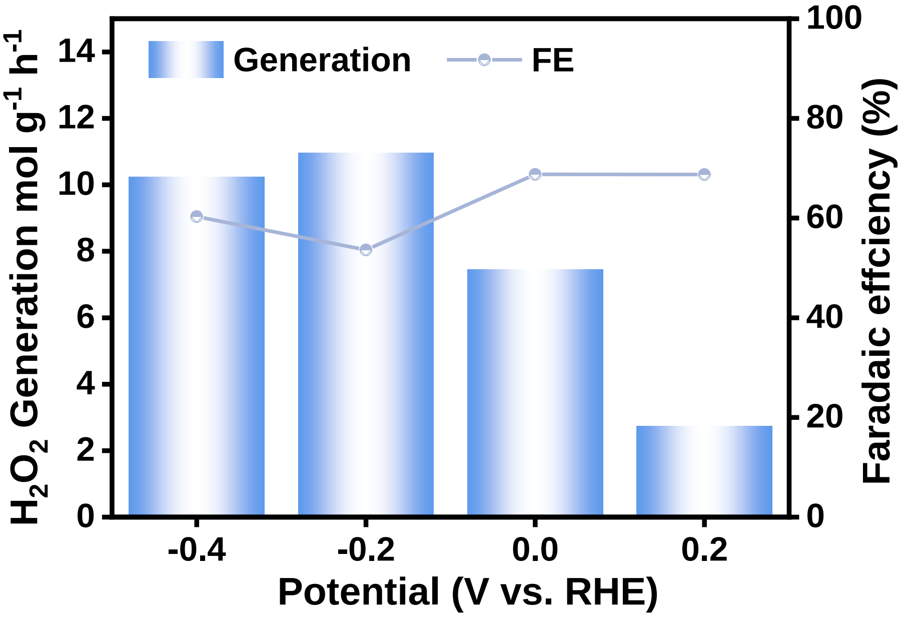

Figure S24. The yield and FE of H_2O_2 for the P-NMG-10 in 0.9 wt.% NaCl.

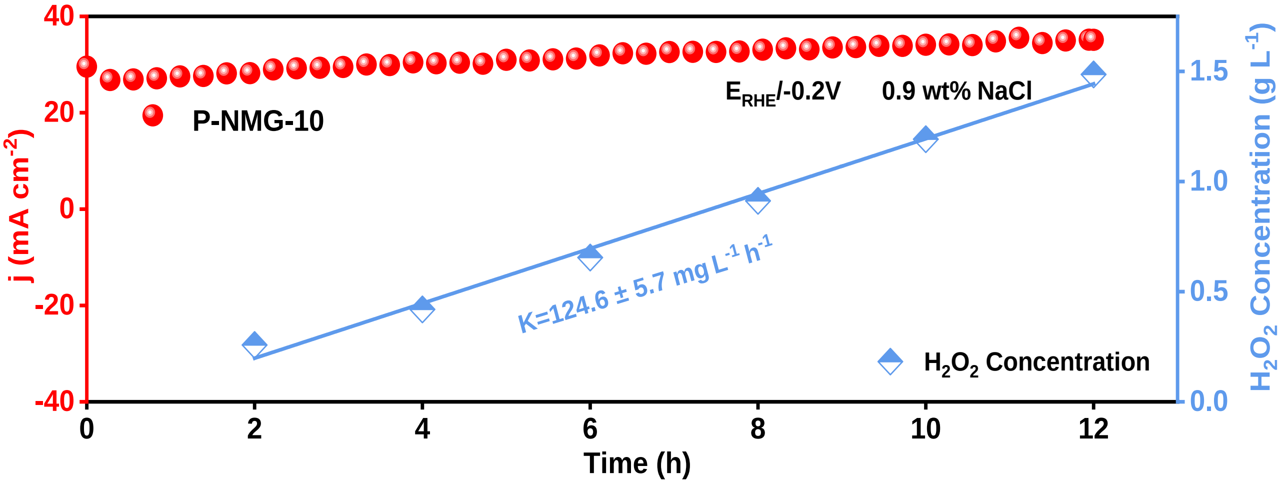

Figure S25. The current density for 24 h in 0.9 wt.% NaCl and linear fitted lines for the change in H_2O_2 concentration.

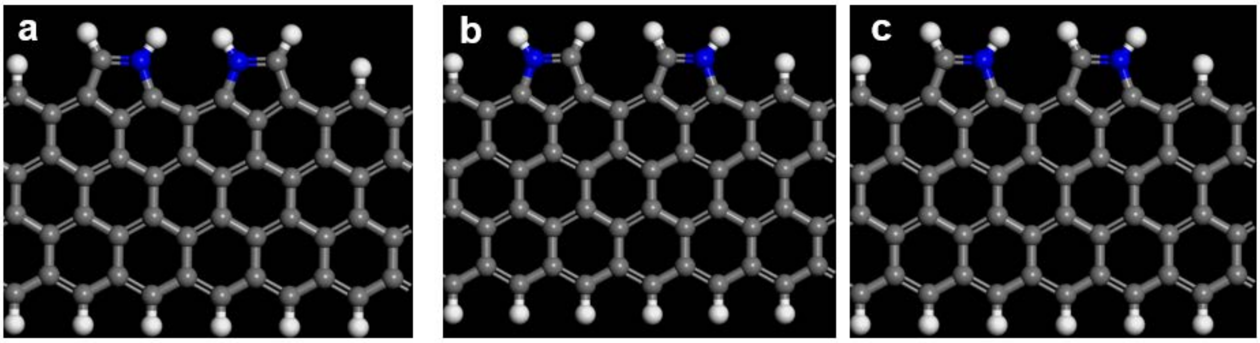

Figure S26. a-c Dual-pyrrolic nitrogen active sites with different structures.

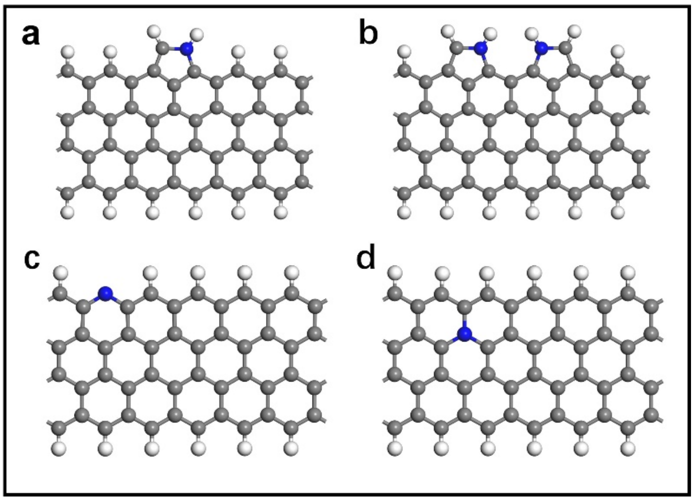

Figure S27. Schematic diagram of N-doped graphene with pyrrolic nitrogen **a-b**, pyridinic nitrogen **c**, and quaternary nitrogen in the bulk phase **d**, respectively.

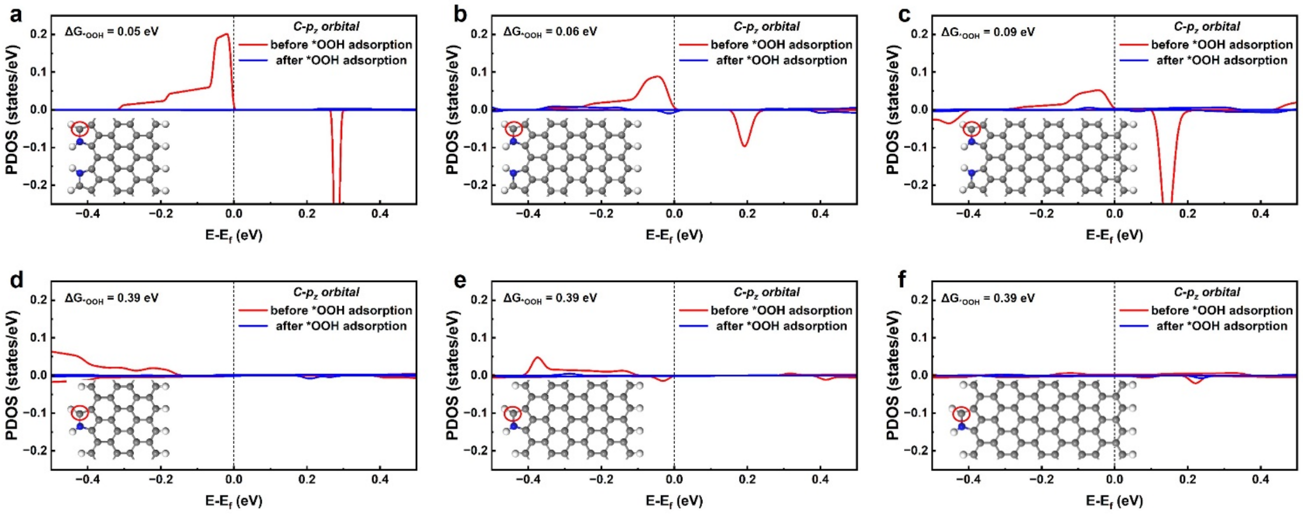

Fig S28 a-c PDOS of p_z orbital of C atom on dual-pyrrolic nitrogen carbon with different nanoribbon widths before and after *OOH adsorption. **d-f** PDOS of p_z orbital of C atom on pyrrole with different nanoribbon widths before and after *OOH adsorption.

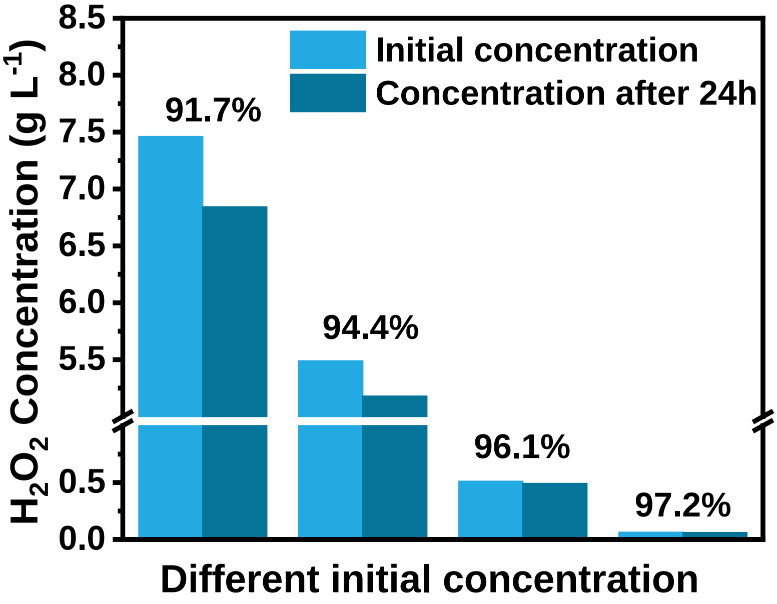

Figure S29. The retention rate of different concentrations of H_2O_2 in 0.1M KOH after 24 h resting.

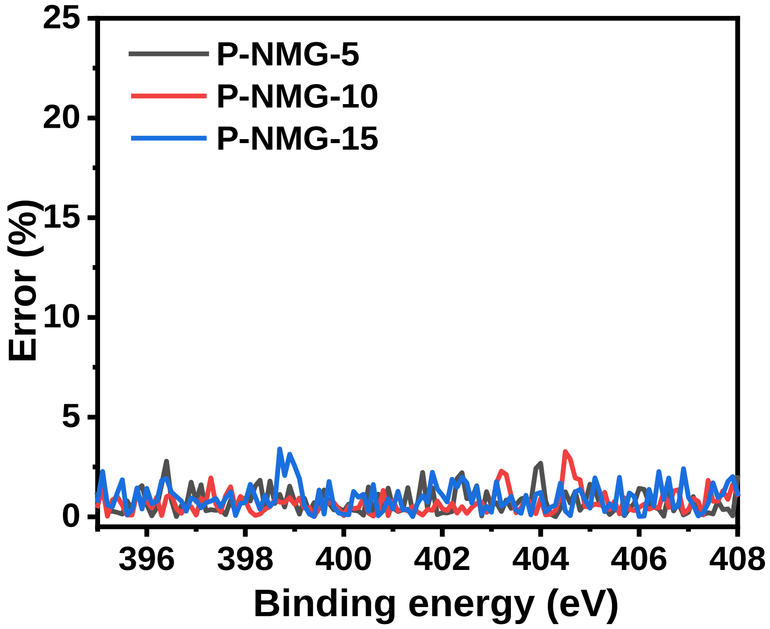

Figure S30 N1s peak fitting error of P-NMG-5/10/15.

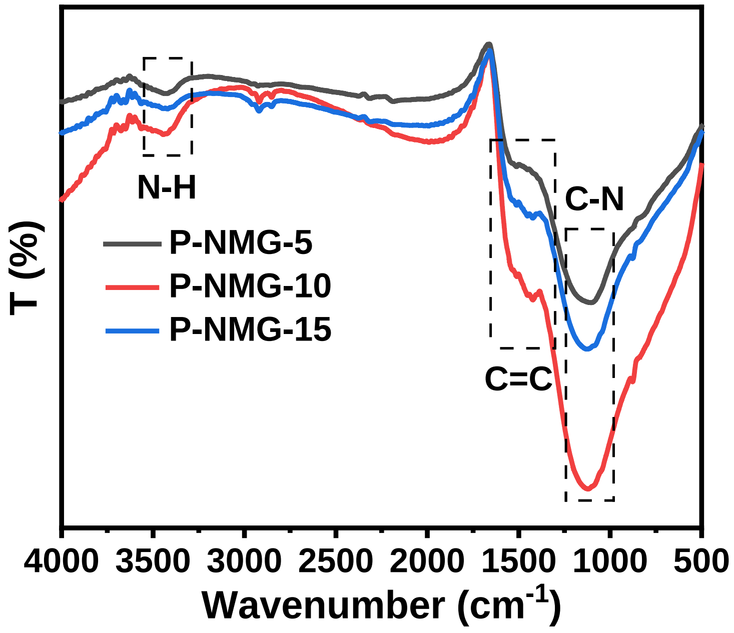

Figure S31 FTIR spectra of the P-NMG-5/10/15.

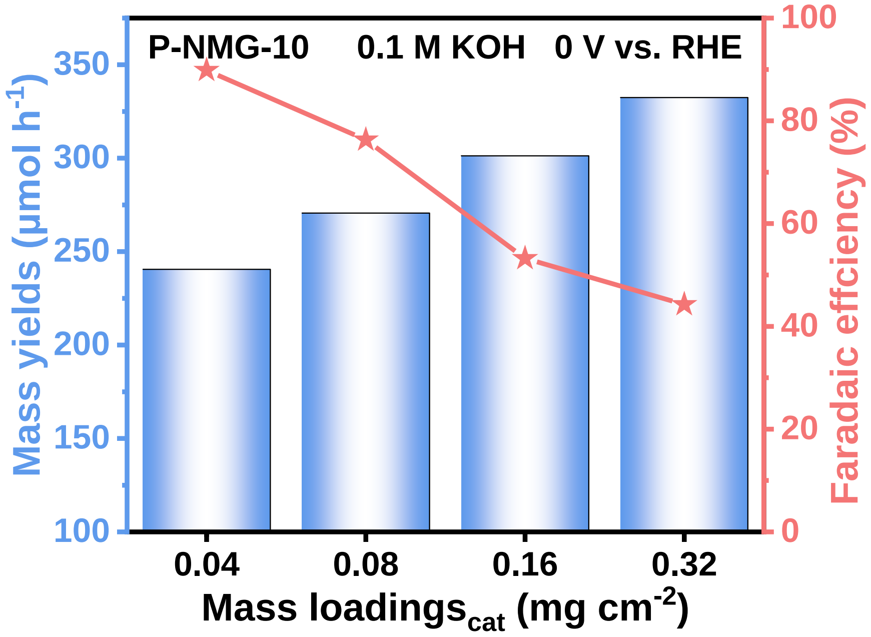

Figure S32 Mass yields for different catalyst loadings per unit electrode area.

With the increase of areal catalyst loading, the H_2O_2 mass yield increases, but the Faradaic efficiency decreases. This may be attributed to the thickened catalyst coating on the carbon paper electrode, and the catalyst beneath the surface layer cannot effectively contact oxygen, leading to the

unfavorable H_2O_2RR that consumes the produced H_2O_2. Considering these results, we believe that an areal loading of 0.04 mg cm^{-2} should be the optimal loading for the preparation of H_2O_2 using this material.

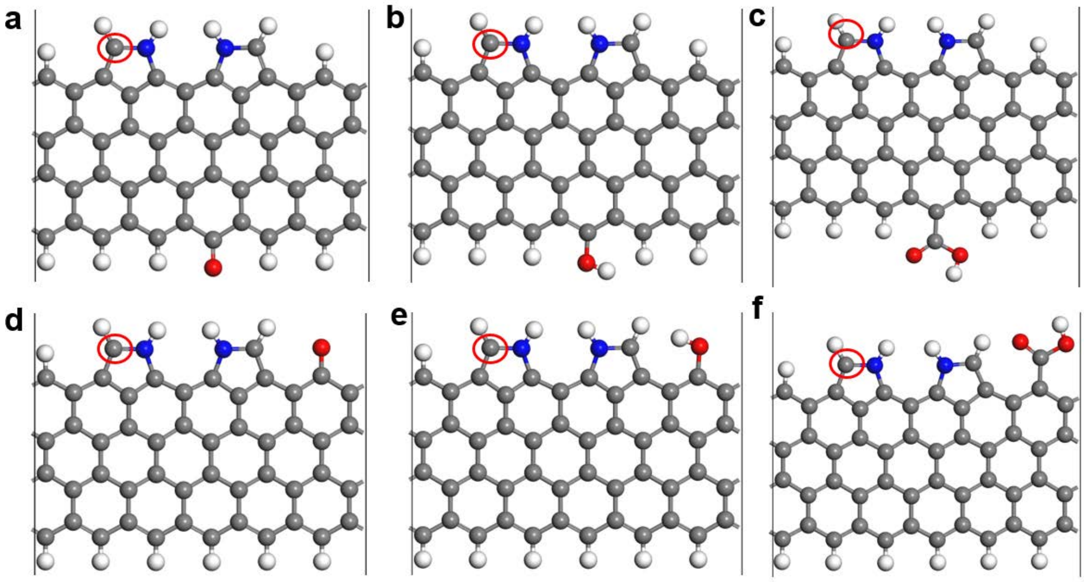

Figure S33. a-c Dual-PyrN-Gr active sites with different oxygen functional groups on the same side of the nitrogen atom. **d-f** Dual-PyrN-Gr active sites with different oxygen functional groups on the opposite side of the nitrogen atom.

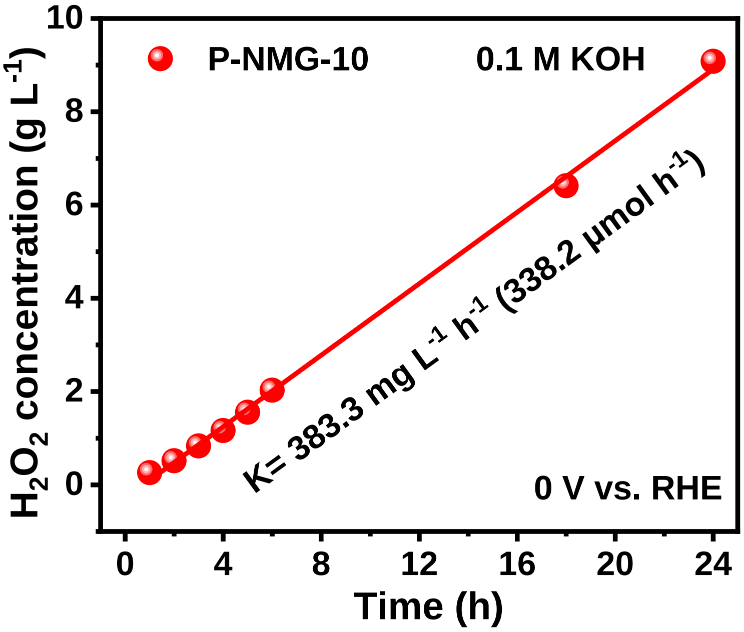

Figure S34 24h non-stop cumulative H_2O_2 concentration test in a flow-cell.

Table S1. Atomic content of C−OH, C−O−C/O=C−O and C=O for P-NMG-0, P-NMG-5, P-NMG-10 and P-NMG-15 according to XPS survey spectra.

Sample	C−OH	C−O−C/O=C−O	C=O
P-NMG-0	9.4	50.1	40.5
P-NMG-5	23.3	41.3	35.4
P-NMG-10	18.9	32.2	48.9
P-NMG-15	19.8	37.9	42.3

Table S2. The electrochemical performance comparison with electrocatalysts recently reported.

Catalysts	Reaction conditions	FE	H_2O_2 yield (mol g^{-1} h^{-1})	Ref.
P-NMG-10	0 V vs. RHE	90	12	This work
P-NMG-10	−0.6 V vs. RHE	80	30	This work
G-COF-950	0.1 V vs. RHE	69.8	1.29	1
N-doped porous carbon	0.1 V vs. RHE	55	0.46	2
N-doped porous carbon	0.2 V vs. RHE	58	0.35	2
N-doped mesoporous carbon	0.1 V vs. RHE	70	0.56	3
Biomass derived N/C	−0.67 V vs. RHE	51	0.05	4
a-NiO NSs	0.51 V vs. RHE	85	0.23	5
Co_1-NG(O)	50 mA cm^{-1}	95.6	0.42	6
Oxo-G/$NH_3 \cdot H_2O$	0.2 V vs. RHE	43.6	0.22	7
rGO-PEI	0.74 V vs. RHE	90.7	0.11	8
Ni-N_2O_2	70 mA cm^{-1}	90	5.9	9
N-FLG-8	1.8 V (cell voltage)	90	9.66	10
Single site Co-NC	50 mA cm^{-1}	52	0.19	11
N-O-P-C-800	0.25 V vs. RHE	65	1.47	12

Note that any value below the theoretical potential for $2e^-$-ORR, i.e., ~0.7 V vs. RHE, can initiate $2e^-$-ORR. For Ref. 8, the testing potential (0.74 V vs. RHE) is just slightly higher than the theoretical

onset potential for 2e^{-}-ORR (i.e., 0.7 V vs. RHE) and this might be due to a highly facile ORR kinetics with negligible overpotential for O_2-to-H_2O_2 conversion.

Table S3. ICP of P-NMG-10

Element	Concentration	Unit	Element	Concentration	Unit
Ag	0.0099	mg/kg	Nd	0.0112	mg/kg
Al	91.5934	mg/kg	Ni	66.5440	mg/kg
As	0.2911	mg/kg	Os	0.0799	mg/kg
Au	0.0106	mg/kg	P	1616.5527	mg/kg
B	0.2410	mg/kg	Pb	0.0930	mg/kg
Ba	0.4477	mg/kg	Pd	0.0541	mg/kg
Be	0.0001	mg/kg	Pr	0.0277	mg/kg
Bi	0.2592	mg/kg	Pt	0.5546	mg/kg
Ca	4499.6769	mg/kg	Rb	0.3392	mg/kg
Cd	0.0114	mg/kg	Re	0.2399	mg/kg
Ce	0.0075	mg/kg	Rh	0.0238	mg/kg
Co	0.0325	mg/kg	Ru	0.1644	mg/kg
Cr	0.2281	mg/kg	S	162.5602	mg/kg
Cu	2.6291	mg/kg	Sb	0.9527	mg/kg
Dy	0.1747	mg/kg	Sc	0.0225	mg/kg
Er	0.0479	mg/kg	Se	0.4832	mg/kg
Eu	0.0003	mg/kg	Si	61.8932	mg/kg
Fe	12.6863	mg/kg	Sm	0.0216	mg/kg
Ga	0.0406	mg/kg	Sn	0.2333	mg/kg
Gd	0.0296	mg/kg	Sr	0.9741	mg/kg
Ge	0.6928	mg/kg	Ta	0.1813	mg/kg
Hf	0.0560	mg/kg	Tb	0.1122	mg/kg
Hg	0.4056	mg/kg	Te	0.9128	mg/kg
Ho	0.0784	mg/kg	Th	0.3039	mg/kg
In	0.0952	mg/kg	Ti	3.8156	mg/kg
Ir	0.1616	mg/kg	Tl	0.3653	mg/kg
K	153.4270	mg/kg	Tm	0.0055	mg/kg
La	0.0323	mg/kg	U	0.5785	mg/kg
Li	3.9857	mg/kg	V	0.1359	mg/kg
Lu	0.0087	mg/kg	W	0.3043	mg/kg
Mg	175.6201	mg/kg	Y	0.0035	mg/kg
Mn	125.8709	mg/kg	Yb	0.0021	mg/kg
Mo	0.1651	mg/kg	Zn	0.7164	mg/kg
Na	6544.9585	mg/kg	Zr	0.0152	mg/kg
Nb	0.0953	mg/kg			

Table S4. Formation energy and overpotential of active sites with different oxygen functional groups.

Active sites with different oxygen functional groups	Formation energy (eV)	Overpotential (V)
C=O（Same side as N atom）	−520.17	0.16
C=O（Opposite side as N atom）	−520.74	0.06
COH（Same side as N atom）	−524.21	0.01
COH（Opposite side as N atom）	−524.24	0.03
COOH（Same side as N atom）	−539.7	0.46
COOH（Opposite side as N atom	−540.05	0.05

Supplementary References:

1. Zhang, Junyu, *et al*. Graphitic N in Nitrogen-Doped Carbon Promotes Hydrogen Peroxide Synthesis from Electrocatalytic Oxygen Reduction. *Carbon* **163**, 154-161 (2020).

2. Sun, Yanyan, *et al*. Structure, Activity, and Faradaic Efficiency of Nitrogen-Doped Porous Carbon Catalysts for Direct Electrochemical Hydrogen Peroxide Production. *ChemSusChem* **11**, 3388-3395 (2018).

3. Sun, Yanyan, *et al*. Efficient Electrochemical Hydrogen Peroxide Production from Molecular Oxygen on Nitrogen-Doped Mesoporous Carbon Catalysts. *ACS Catal.* **8**, 2844-2856 (2018).

4. Yang, Yiran, *et al*. A Biomass Derived N/C-Catalyst for the Electrochemical Production of Hydrogen Peroxide. *Chem. Commun.* **53**, 9994-9997 (2017).

5. Li, Ruilong, *et al*. Short-range Order in Amorphous Nickel Oxide Nanosheets Enables Selective and Efficient Electrochemical Hydrogen Peroxide Production. *Cell Rep. Phys. Sci.* **3**, 100788 (2022).

6. Jung, Euiyeon, *et al*. Atomic-Level Tuning of Co-N-C Catalyst for High-Performance Electrochemical H_2O_2 Production. *Nat. Mater.* **19**, 436-442 (2020).

7. Han, Lei, *et al*. In-Plane Carbon Lattice-Defect Regulating Electrochemical Oxygen Reduction to Hydrogen Peroxide Production over Nitrogen-Doped Graphene. *ACS Catal.* **9**, 1283-1288 (2019).

8. Xiao, Xue, *et al*. Enhancing the Selectivity of H_2O_2 Electrogeneration by Steric Hindrance Effect. *ACS Appl. Mater. Inter.* **10**, 42534-42541 (2018).

9. Wang, Yulin, *et al*. High-Efficiency Oxygen Reduction to Hydrogen Peroxide Catalyzed by Nickel Single-Atom Catalysts with Tetradentate N_2O_2 Coordination in A Three-phase Flow Cell. *Angew. Chem. Int. Ed.* **59**, 13057-13062 (2020).

10. Li, Laiquan, *et al*. Tailoring selectivity of electrochemical hydrogen peroxide generation by tunable pyrrolic-nitrogen-carbon. *Adv. Energy Mater.* **10**, 2000789 (2020).

11. Sun, Yanyan, *et al*. Activity-Selectivity Trends in The Electrochemical Production of Hydrogen Peroxide over Single-Site Metal-Nitrogen-Carbon Catalysts. *J. Am. Chem. Soc.* **141**, 12372-12381 (2019).

12. Zhang, H., *et al*. Electrocatalyst Derived from Fungal Hyphae and its Excellent Activity for Electrochemical Production of Hydrogen Peroxide. *Electrochim. Acta* **308**, 74-82 (2019).
